# PLK1 and FoxM1 expressions positively correlate in papillary thyroid carcinoma and their combined inhibition results in synergistic anti‐tumor effects

**DOI:** 10.1002/1878-0261.13610

**Published:** 2024-02-15

**Authors:** Pratheesh Kumar Poyil, Abdul K. Siraj, Divya Padmaja, Sandeep Kumar Parvathareddy, Saravanan Thangavel, Khadija Alobaisi, Roxanne Diaz, Rafia Begum, Wael Haqawi, Saif S. Al‐Sobhi, Fouad Al‐Dayel, Khawla S. Al‐Kuraya

**Affiliations:** ^1^ Human Cancer Genomic Research King Faisal Specialist Hospital and Research Center Riyadh Saudi Arabia; ^2^ Department of Surgery King Faisal Specialist Hospital and Research Centre Riyadh Saudi Arabia; ^3^ Department of Pathology King Faisal Specialist Hospital and Research Centre Riyadh Saudi Arabia

**Keywords:** apoptosis, FoxM1, papillary thyroid cancer, PLK1, stemness

## Abstract

Polo‐like kinase 1 (PLK1; also known as serine/threonine‐protein kinase PLK1) serves as a central player in cell proliferation, exerting critical regulatory roles in mitotic processes and cell survival. We conducted an analysis of PLK1 protein expression in a large cohort of samples from papillary thyroid carcinoma (PTC) patients and examined its functional significance in PTC cell lines, both *in vitro* and *in vivo*. *PLK1* overexpression was noted in 54.2% of all PTC and was significantly associated with aggressive clinicopathological parameters; it was also found to be an independent prognostic marker for shorter recurrence‐free survival. Given the significant association between PLK1 and forkhead box protein M1 (FoxM1), and their concomitant overexpression in a large proportion of PTC samples, we explored their correlation and their combined inhibitions in PTC *in vitro* and *in vivo*. Inhibition of PLK1 expression indeed suppressed cell proliferation, leading to cell cycle arrest and apoptosis in PTC cell lines. Significantly, the downregulation of PLK1 reduced the self‐renewal capability of spheroids formed from PTC cells. Immunoprecipitation analysis shows that PLK1 binds to FoxM1 and vice versa *in vitro*. Mechanistically, *PLK1* knockdown suppresses FoxM1 expression, whereas inhibition of *FoxM1* does not affect PLK1 expression, which suggests that PLK1 acts through the FoxM1 pathway. The combined treatment of a PLK1 inhibitor (volasertib) and a FoxM1 inhibitor (thiostrepton) demonstrated a synergistic effect in reducing PTC cell growth *in vitro* and delaying tumor growth *in vivo*. This study highlights the important role of PLK1 in PTC tumorigenesis and prognosis. It also highlights the synergistic therapeutic potential of dual‐targeting PLK1 and FoxM1 in PTC, unveiling a potential innovative therapeutic strategy for managing aggressive forms of PTC.

## Graphical abstract

The authors examined the prognostic relevance of PLK1 in papillary thyroid carcinoma (PTC) patients, studying its role in PTC cell lines *in vitro* and *in vivo*. PLK1 overexpression in PTC correlates significantly with aggressive clinicopathological features. Interestingly, PLK1 shows a physical association with FoxM1, and the combined inhibition of these proteins demonstrates a synergistic anti‐tumor effect in both *in vitro* and *in vivo* settings.

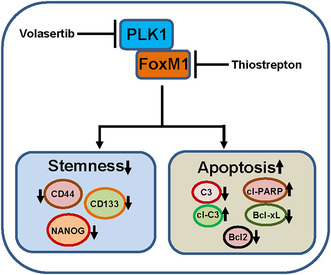

**Abbreviations**
ATCC: American‐Type Culture Collection
Bcl‐2: B‐cell lymphoma 2
Bcl‐xL: B‐cell lymphoma‐extra large
CDK1: cyclin‐dependent kinase 1
DMEM: Dulbecco's Modified Eagle Medium
DSMZ: Deutsche Sammlung von Mikroorganismen und Zellkulturen GmbH
EMT: epithelial to mesenchymal transition
FoxM1: forkhead box protein M1
IHC: immunohistochemistry
PARP: poly (ADP‐ribose) polymerase
PLK1: Polo‐like kinase 1
PTC: papillary thyroid carcinoma
TERT: telomerase reverse transcriptase
TMA: tissue microarray

## 1 Introduction

Papillary thyroid carcinoma accounts for more than 90% of endocrine malignancies [1]. Despite favorable outcomes following standard treatment with surgery and radioiodine (RAI) therapy, a small proportion may develop recurrence and metastases [2]. Predictive biomarkers are needed to be identified and develop effective combinatorial treatment approaches. Mammalian Polo‐like kinase 1 (PLK1) is a serine/threonine kinase with significant roles in regulating cell proliferation and serving as a crucial regulator for the progression through the G2‐M phase of the cell cycle [3, 4]. PLK1 is also known for regulating centrosome mutation [5]. PLK1 is overexpressed in several cancers, including anaplastic thyroid carcinoma [6], gastric [7], breast [8], neuroblastoma [9], and ovarian cancer [10], where high PLK1 expression is correlated with unfavorable prognostic outcomes [9, 11]. These investigations indicate that targeting PLK1 holds great promise as a valuable therapeutic strategy for cancer treatment.

A recent study indicates that PLK1 is involved in differentiated thyroid cancer (DTC) [12], where PLK1 was shown to be up‐regulated in DTC and pharmacological inhibition has reduced cells in the s‐phase and increased cells in G2/M phase, and induced apoptosis in DTC cell lines [12]. FoxM1, a member of the Fox transcription factor family, plays a crucial role in the regulation of cell cycle progression via activating various genes pivotal for G1 to S and G2 to M phase transition including PLK1 [13, 14]. Interestingly, PLK1 interacts directly with FoxM1 and phosphorylates it, leading to the complete transcription activation of FoxM1 and the formation of a feedback loop [14, 15, 16].

Several studies have highlighted the important role of FoxM1 in tumor pathogenesis, tumor progression, invasion, and metastasis in a variety of cancers including thyroid cancer, breast cancer, ovarian cancer, and others [17, 18, 19]. Moreover, overexpression of FoxM1 is correlated with aggressive behavior, drug resistance, cancer phenotype, and poor outcome [19, 20, 21, 22, 23, 24]. The aforementioned information lays the scientific premise for this study, where our hypothesis posits a potential correlation between PLK1 and FoxM1 in a large Middle Eastern PTC cohort. Moreover, exploring the simultaneous inhibition of PLK1 and FoxM1, both *in vitro* and *in vivo*, may establish a rationale for considering the combined inhibition of these two factors as a potential therapeutic approach for managing PTC.

## 2 Materials and methods

### 2.1 Patient selection and clinico‐pathological data

One thousand seven‐hundred and sixteen PTC patients diagnosed between 1988 and 2020 at King Faisal Specialist Hospital and Research Centre (Riyadh, Saudi Arabia) were included in the study. Diagnosis of PTC was based on clinical history and confirmed by fine needle aspiration cytology. Table 1 summarizes the baseline clinico‐pathological data, collected from patient medical records. Eighth edition of American Joint Committee on Cancer (AJCC) staging system was used for PTC staging [25]. Recurrence was defined as any newly detected tumor (local or distant) or metastatic lymph node based on ultrasound and/or imaging studies in patients who had been previously free of disease following initial treatment.

**Table 1:** Clinico‐pathological characteristics of papillary thyroid carcinoma cohort.

	No.	%
No. of patients	1716	
Age (years)		
Median (range)	38.5 (6.0–87.6)	
≤ 55	319	18.6
> 55	1397	81.4
Sex		
Female	1300	75.7
Male	416	24.3
Histology type		
Classical variant	1080	63.0
Follicular variant	301	17.5
Tall‐cell variant	180	10.5
Other variants	155	9.0
Tumor laterality		
Unilateral	1158	67.5
Bilateral	558	32.5
Tumor focality		
Unifocal	851	49.6
Multifocal	865	50.4
Extrathyroidal extension		
Absent	1006	58.6
Present	710	41.4
Lymphovascular invasion		
Absent	1246	72.6
Present	470	27.4
pT		
T1	688	40.1
T2	555	32.3
T3	344	20.0
T4	125	7.3
Unknown	4	0.3
Lymph node metastasis		
Absent	716	41.7
Present	831	48.4
Unknown	169	9.9
Distant metastasis		
Absent	1568	91.4
Present	148	8.6
Stage		
I	1450	84.5
II	182	10.6
III	24	1.4
IV	53	3.1
Unknown	7	0.4
*BRAF* mutation		
Present	934	54.4
Absent	744	43.4
Unknown	38	2.2
*TERT* mutation		
Present	207	12.1
Absent	1393	81.2
Unknown	116	6.7
Recurrence		
Yes	307	17.9
No	1409	82.1

### 2.2 Ethics declarations

Institutional Review Board of King Faisal Specialist Hospital and Research Centre provided ethical approval for the current study (RAC# 2110 031, RAC# 2220 002 and 2211 168). Research Advisory Council (RAC) granted waiver of informed consent for use of retrospective patient case data. All the methods were carried out in accordance with the Declaration of Helsinki.

### 2.3 *BRAF* and *TERT* mutation analysis

*BRAF* and *TERT* mutation data were assessed in our laboratory by Sanger sequencing and has been published by us previously [26, 27, 28]. *BRAF* mutation analysis was performed in 1678 cases and *TERT* mutation analysis was done in 1600 cases.

### 2.4 Tissue microarray construction and immunohistochemistry analysis

Tissue microarray (TMA) format was utilized for immunohistochemical (IHC) analysis of the PTC samples. TMA was constructed as previously described [29]. Briefly, modified semiautomatic robotic precision instrument (Beecher Instruments, Woodland, WI, USA) was used to punch tissue cylinders with a diameter of 0.6 mm from representative tumor area of the donor tissue block and brought into the recipient paraffin block. Two cores of PTC were arrayed from each case.

Tissue microarray slides were processed and stained manually as described previously [30]. Primary antibodies against PLK1 (mouse monoclonal, ab‐17 056, 1 : 500, pH 9.0; Abcam, Cambridge, UK) and FoxM1 (clone K‐19, 1 : 2500, pH 9.0; Santa Cruz Biotechnology, Santa Cruz, CA, USA). The Dako Envision Plus System kit (Glostrup, Denmark) was used as the secondary detection system with 3, 30‐diaminobenzidine as chromogen. All slides were counter stained with hematoxylin, dehydrated, cleared, and mounted. Negative controls included omission of the primary antibody. Normal tissues of different organ system were also included in the TMA to serve as control. Only fresh‐cut slides were stained simultaneously to minimize the influence of slide aging and maximize reproducibility of the experiment.

PLK1 staining was scored using immunoreactivity score (IRS), as described previously [31]. Briefly, staining intensity was scored as 0: negative; 1: weak; 2: moderate; or 3: strong, and staining proportion was scored as 0, 0%; 1, 1–10%; 2, 11–50%; 3, 51–80%; or 4, more than 80% positive cells. The final IRS score was calculated by multiplying the intensity and proportion scores. Low expression of PLK1 was defined as IRS 0–6 and high expression of PLK1 was defined as IRS more than 6. FoxM1 staining was scored using *H* score, as described previously [18]. x‐tile software [32] was used to define the optimal cut‐off score. Based on x‐tile plots, PTC cases with complete absence (*H* score = 0) was defined as no expression and the other group (*H* score ≥ 1) was defined as overexpression for FoxM1.

### 2.5 Cell culture

Nthy‐ori 3–1 (RRID:CVCL_2659), normal human primary thyroid follicular epithelial cell line was obtained from Sigma‐Aldrich (St. Louis, MO, USA). The PTC cell line, BCPAP (RRID:CVCL_0153), was purchased from DSMZ (Braunschweig, Germany), and TPC‐1 (RRID:CVCL_6298) was kindly provided by B. McIver (Department of Endocrinology, Mayo Clinic, Rochester, MN, USA). K1 cell line (RRID:CVCL_2537) was purchased from American Type Culture Collection (ATCC, Manassas, VA, USA). Cell lines were cultured using RPMI 1640 media supplemented with 10% fetal bovine serum (FBS), along with 100 Units·mL^−1^ of penicillin/streptomycin and 100 Units·mL^−1^ of Glutamine, following previously established protocols [18]. These cell lines were tested negative for mycoplasma and their authentication was conducted internally using short tandem repeats PCR, and the obtained results were consistent with previously published data [26, 33].

### 2.6 Reagents and antibodies

The PLK1 inhibitor, volasertib, was purchased from Selleck Chemicals (Houston, TX, USA). Antibodies against PLK1 (4513), Bcl‐2 (2876), Bcl‐xl (2762), cleaved caspase‐3 (9664), PARP (9542), CD44 (3570), CD133 (64326), NANOG (4903), Cyclin B1 (4135), phospho‐Bad (9295), Bad (9292) and GAPDH (5174) were purchased from Cell Signaling Technology (Danvers, MA, USA). Antibodies against FoxM1 (sc‐376 471) and caspase‐3 (sc‐56 053) were purchased from Santa Cruz Biotechnology, Inc. Cyclin D1 (ab139260) was obtained from Abcam (Cambridge, MA, USA). Phospho‐CDK1/CDC2 (orb127843) was purchased from Biorbyt Ltd (Cambridge, UK).

### 2.7 Gene silencing using siRNA

*FoxM1* siRNA and scrambled control siRNA were purchased from Qiagen (Valencia, CA, USA). Cells were transfected using Lipofectamine 2000 (Invitrogen, Carlsbad, CA, USA) for a duration of 6 h, after which the lipid‐siRNA complex was removed, and fresh growth medium supplemented with 20% fetal bovine serum was added. Following a 48‐h post‐transfection period, cells were harvested and utilized for various experiments.

### 2.8 Plasmid and transfection

Plasmid DNA encoding human *PLK1* and shRNA targeting human *PLK1* were purchased from Origene (Rockville, MD, USA). The forced expression and knockdown of *PLK1* in Nthy‐ori 3–1 cell line and PTC cell lines, respectively, were performed using Lipofectamine™2000 (Invitrogen) according to the manufacturer's protocol. In brief, cells were initially seeded in 6‐well culture plates. When they reached approximately 50% confluence, transfection was carried out using 4 μg of plasmid. After a 48‐h post‐transfection period, stable clones with overexpressed *PLK1*, resistant to G418, and stable knockdown clones, resistant to puromycin, were isolated. The confirmation of successful overexpression and knockdown of PLK1 protein production was validated through immunoblotting.

### 2.9 Sphere forming assay

Cells, at a density of 500 cells per well, were seeded in Corning 24‐well ultra‐low attachment plates (Sigma‐Aldrich). These cells were cultured in serum‐free DMEM‐F12 (ATCC) medium, which was supplemented with B27 (Thermo Fisher Scientific, Grand Island, NY, USA), 20 ng·mL^−1^ EGF (Sigma‐Aldrich), 0.4% BSA (Sigma‐Aldrich), and 4 μg·mL^−1^ insulin (Sigma‐Aldrich). Fresh medium was added every 2 days. Spheroids were counted and photographed on day 14. To assess secondary spheroid formation, the primary spheroids were dissociated into single cells and subsequently cultured in 24‐well ultra‐low attachment plates, using spheroid culture medium, for an additional 10 days.

### 2.10 Animals and xenografts study

Six‐week‐old female nude mice (NU/J) were procured from Jackson Laboratories (Bar Harbor, ME, USA) and were housed in a sterile and pathogen‐free environment (12 h light/12 h darkness) with food and water *ad libitum* at least 1 week before use. All animal experiments were conducted in strict adherence to institutional guidelines and were approved by the Animal Care and Use Committee (ACUC) of King Faisal Specialist Hospital and Research Center on 24 October 2022 (RAC#2220002). For the xenograft study, TPC‐1 cells (4 × 10^6^ cells per mouse) were resuspended in serum‐free medium combined with matrigel basement membrane matrix at a 1 : 1 ratio. These cell mixtures were subsequently subcutaneously injected into the flanks of NU/J mice (*n* = 5). Once the tumors reached a size of approximately 100 mm^3^, the mice received intraperitoneal treatments, which included vehicle (0.1% DMSO), volasertib (20 mg·kg^−1^), thiostrepton (20 mg·kg^−1^), and a combination of volasertib and thiostrepton. These treatments were administered twice a week for 30 days. Throughout the study, the body weight and tumor volume of each mouse were closely monitored on a weekly basis. Following 5 weeks of treatment, the mice were euthanized, and individual tumors were weighed before being rapidly snap‐frozen in liquid nitrogen for storage.

### 2.11 Statistical analysis

The associations between clinico‐pathological variables and protein expression were performed using contingency table analysis and Chi square tests. Mantel‐Cox log‐rank test was used to evaluate recurrence‐free survival. Survival curves were generated using the Kaplan–Meier method. Cox proportional hazards regression model was used for multivariate analysis. Two‐sided tests were used for statistical analyses with a limit of significance defined as *P* value < 0.05. Data analysis was performed using the jmp14.0 (SAS Institute, Inc., Cary, NC, USA) software package.

In all functional studies, the data are presented as means ± standard deviation (SD) derived from triplicates in an independent experiment. This experiment was repeated at least two times, yielding consistent results. Statistical significance was assessed using a two‐tailed Student's *t*‐test, with a significance threshold set at *P* < 0.05.

## 3 Results

### 3.1 Patient characteristics

Median age of the study population was 38.5 years (range: 6.0–87.6 years), with a male: female ratio of 1 : 3. 32.5% (558/1716) of tumors were bilateral and 50.4% (865/1716) were multifocal. Extrathyroidal extension was noted in 41.4% (710/1716) of DTCs. Regional lymph node metastasis (LNM) was noted in 48.4% (831/1716) of cases and distant metastasis was present in 8.6% (148/1716). Frequency of *BRAF* and *TERT* mutations was 54.4% (934/1716) and 12.1% (207/1716), respectively (Table 1).

### 3.2 PLK1 expression in papillary thyroid cancer and its clinico‐pathological associations

**Fig. 1:**
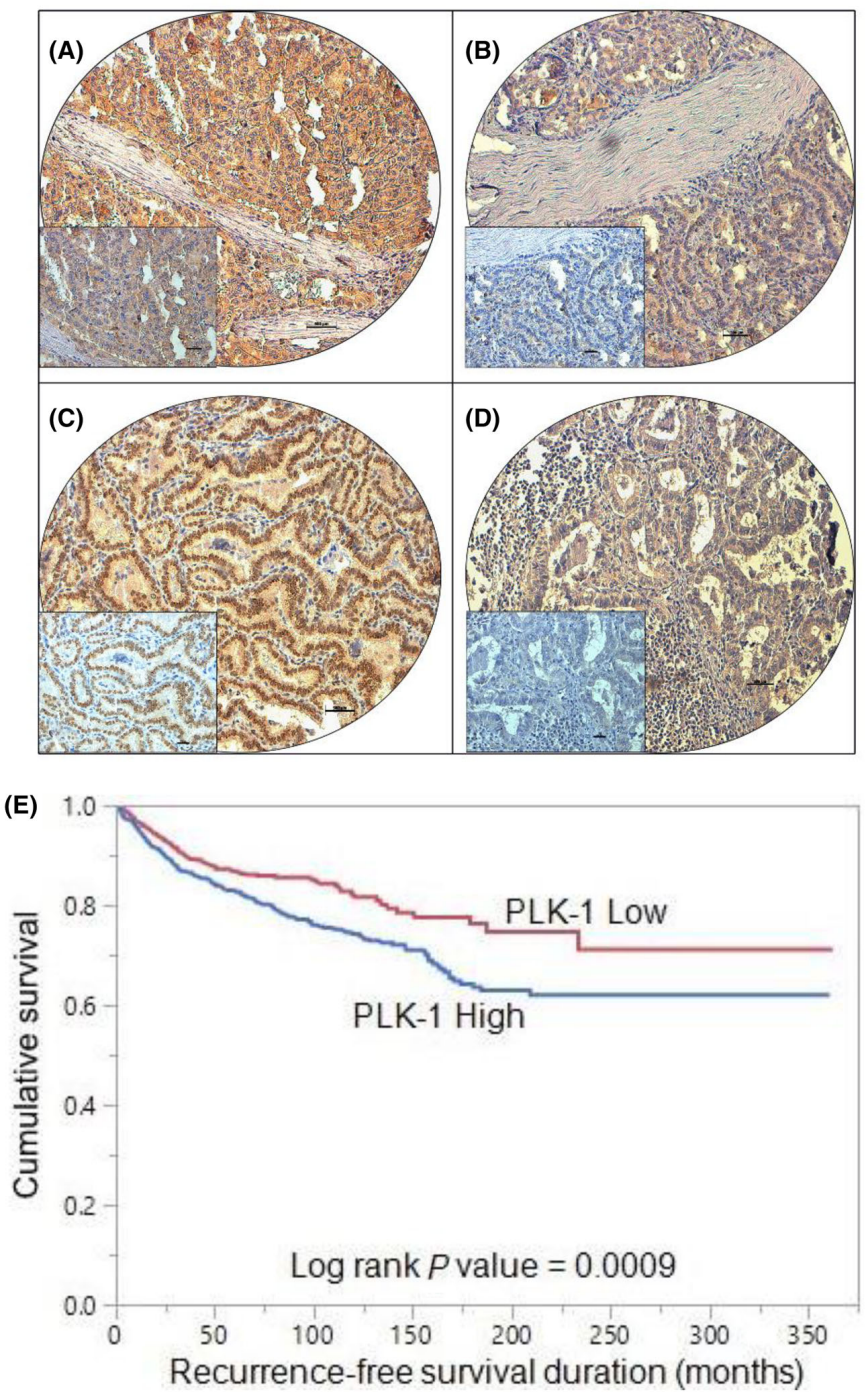
Tissue microarray (TMA) based immunohistochemistry analysis of PLK1 and FoxM1 in papillary thyroid cancer (PTC) patients. PTC TMA spots showing overexpression of PLK1 (A) and FoxM1 (C). In contrast, another set of TMA spots showing reduced expression of PLK1 (B) and FoxM1 (D). 20×/0.70 objective on an Olympus BX 51 microscope (Olympus America Inc, Center Valley, PA, USA) with the inset showing a 40× 0.85 aperture magnified view of the same TMA spot (scale bar = 500 μm). (E) Recurrence‐free survival (RFS). Kaplan–Meier survival plot showing statistically significant poor RFS in PLK1 high expression cases compared to PLK1 low expression (*P* = 0.0009).

PLK1 protein expression was analyzed immunohistochemically in 1716 PTC samples. However, immunohistochemistry data was interpretable in 1612 samples and hence were included for further analysis. Over‐expression of PLK1 was noted in 54.2% (874/1612) of cases (Fig. 1) and was significantly associated with adverse clinico‐pathological parameters such as extrathyroidal extension (*P* < 0.0001), lymphovasacular invasion (*P* = 0.0021), T4 tumors (*P* < 0.0001), lymph node metastasis (*P* < 0.0001) and tumor recurrence (*P* < 0.0001) (Table 2). We also found a significant association between PLK1 expression and molecular markers like *BRAF* mutation (*P* < 0.0001) and *TERT* mutation (*P* = 0.0021). Interestingly, PLK1 over‐expression was associated with cell cycle regulator, FoxM1 (*P* < 0.0001) in our cohort (Table 2).

**Table 2:** Clinico‐pathological associations of PLK‐1 expression in papillary thyroid carcinoma.

	Total		PLK‐1 high		PLK‐1 low		*P* value
	No.	%	No.	%	No.	%	
No. of patients	1612		874	54.2	738	45.8	
Age (years)							
≤ 55	1316	81.6	700	80.1	616	83.5	0.0810
> 55	296	18.4	174	19.9	122	16.5	
Sex							
Female	1227	76.1	666	76.2	561	76.0	0.9308
Male	385	23.9	208	23.8	177	24.0	
Histology type							
Classical variant	1018	63.1	598	68.4	420	56.9	< 0.0001
Follicular variant	287	17.8	100	11.4	187	25.3	
Tall‐cell variant	167	10.4	103	11.8	64	8.7	
Other variants	140	8.7	73	8.4	67	9.1	
Tumor laterality							
Unilateral	1083	67.2	575	65.8	508	68.8	0.1945
Bilateral	529	32.8	299	34.2	230	31.2	
Tumor focality							
Unifocal	804	49.9	425	48.6	379	51.4	0.2751
Multifocal	808	50.1	449	51.4	359	48.6	
Extrathyroidal extension							
Absent	948	58.8	472	54.0	476	64.5	< 0.0001
Present	664	41.2	402	46.0	262	35.5	
Lymphovascular invasion							
Absent	1178	73.1	666	76.2	512	69.4	0.0021
Present	434	26.9	208	23.8	226	30.6	
pT							
T1	643	40.0	371	42.5	272	37.0	< 0.0001
T2	514	32.0	264	30.2	250	34.0	
T3	333	20.7	157	18.0	176	24.0	
T4	118	7.3	81	9.3	37	5.0	
Lymph node metastasis							
Absent	680	46.4	331	41.4	349	52.4	< 0.0001
Present	786	53.6	469	58.6	317	47.6	
Distant metastasis							
Absent	1469	91.1	787	90.0	682	92.4	0.0960
Present	143	8.9	87	10.0	56	7.6	
Stage							
I	1364	85.0	723	83.2	641	87.1	0.0981
II	169	10.5	98	11.3	71	9.6	
III	22	1.4	15	1.7	7	1.0	
IV	50	3.1	33	3.8	17	2.3	
Tumor recurrence							
Yes	302	18.7	197	22.5	105	14.2	< 0.0001
No	1310	81.3	677	77.5	633	85.8	
*BRAF* mutation							
Present	881	55.8	531	61.6	350	48.9	< 0.0001
Absent	697	44.2	331	38.4	366	51.1	
*TERT* mutation							
Present	198	13.1	129	15.6	69	10.2	0.0021
Absent	1309	86.9	700	84.4	609	89.8	
FOXM1 IHC							
High	469	29.8	345	40.1	124	17.4	< 0.0001
Low	1102	70.2	515	59.9	587	82.6	

### 3.3 PLK1 expression and clinical outcome

We further analyzed the association of PLK1 over‐expression with clinical outcome in our cohort of PTC. Kaplan–Meier curve analysis showed that patients exhibiting PLK1 over‐expression had a significantly shorter RFS compared to those who had low PLK1 expression (*P* = 0.0009) (Fig. 1E). This association remained significant on multivariate analysis, after adjusting for other clinico‐pathological variables (Hazard ratio = 1.32, 95% confidence interval = 1.02–1.71, *P* = 0.0325), suggesting that PLK‐1 was an independent predictor of RFS (Table 3).

**Table 3:** Univariate and multivariate analysis of PLK‐1 expression using Cox proportional hazard model for recurrence‐free survival.

Clinico‐pathological variables	Recurrence‐free survival			
	Univariate		Multivariate	
	Hazard ratio (95% CI)	*P*‐value	Hazard ratio (95% CI)	*P*‐value
Age				
Above ≥ 55 years (vs. < 55 years)	2.90 (2.27–3.68)	< 0.0001	2.27 (1.70–3.00)	< 0.0001
Sex				
Male (vs. female)	1.77 (1.39–2.23)	< 0.0001	1.36 (1.04–1.76)	0.0244
Histology				
Aggressive variants (vs. non‐aggressive variants)	1.43 (1.04–1.93)	0.0276	1.15 (0.79–1.64)	0.4528
Tumor laterality				
Bilateral (vs. unilateral)	1.67 (1.33–2.09)	< 0.0001	1.18 (0.92–1.52)	0.1973
Tumor focality				
Multifocal (vs. unifocal)	1.23 (0.98–1.54)	0.0722		
Extrathyroidal extension				
Present (vs. absent)	2.94 (2.33–3.74)	< 0.0001	1.60 (1.21–2.14)	0.0010
Lymphovascular invasion				
Present (vs. absent)	1.31 (1.01–1.69)	0.0452	0.96 (0.70–1.30)	0.7791
pT				
T4 (vs. T1–T3)	1.93 (1.52–2.45)	< 0.0001	1.28 (0.98–1.65)	0.0651
Lymph node metastasis				
Present (vs. absent)	2.70 (2.09–3.53)	< 0.0001	2.01 (1.51–2.70)	< 0.0001
Distant metastasis				
Present (vs. absent)	7.23 (5.28–9.69)	< 0.0001	3.55 (2.49–4.96)	< 0.0001
PLK‐1 expression				
High (vs. Low)	1.49 (1.18–1.89)	0.0008	1.32 (1.02–1.71)	0.0325

### 3.4 Inhibition of PLK1 impedes cell proliferation and induces cell cycle arrest and apoptosis

**Fig. 2:**
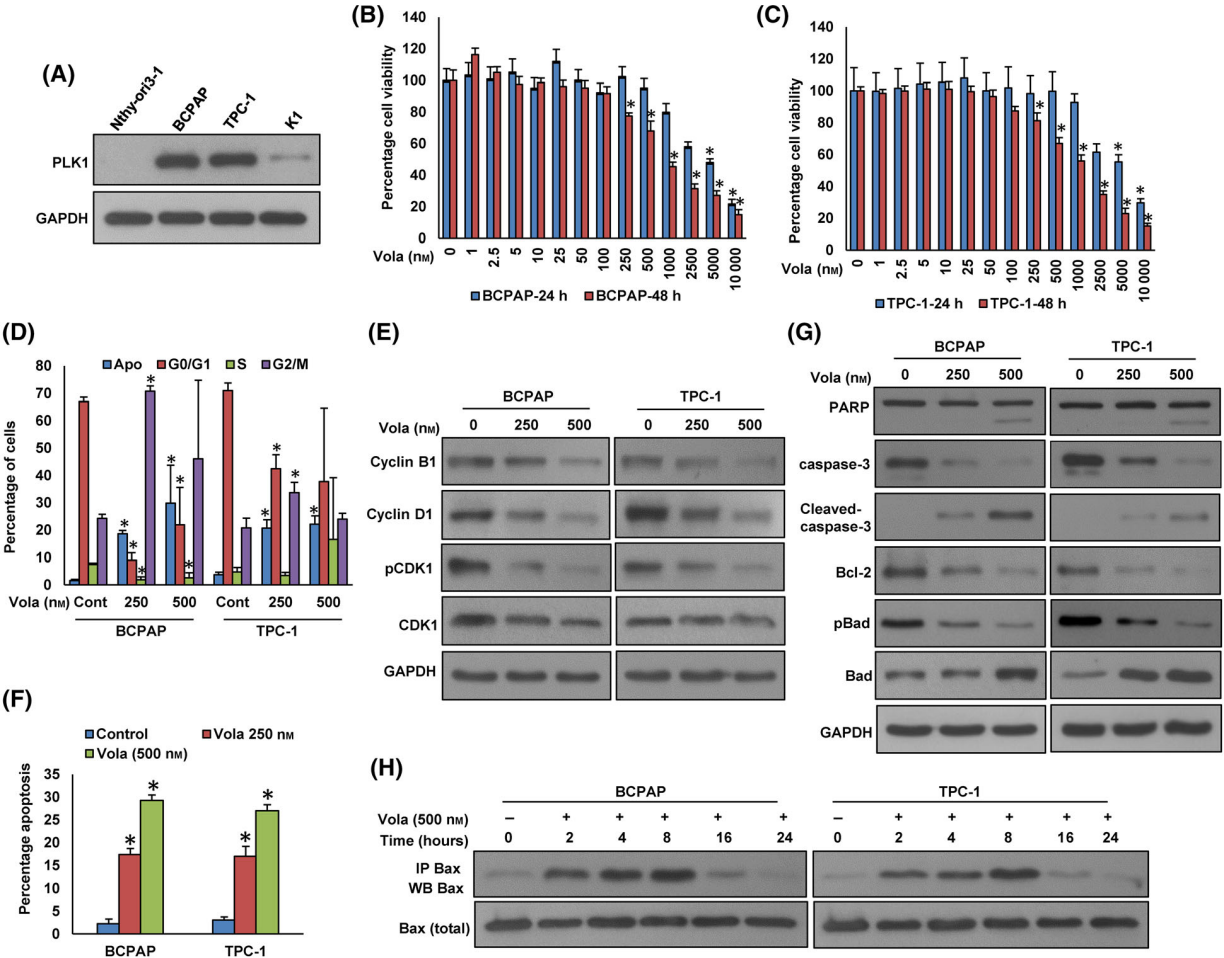
Inhibition of PLK1 impedes cell proliferation and induces cell cycle arrest and apoptosis. (A) Basal expression of PLK1 in Nthy‐ori 3–1 and PTC cell lines. Proteins were extracted from Nthy‐ori 3–1 cell line and three PTC cell lines and subjected to immunoblotting using indicated antibodies (*n* = 3). (B, C) Volasertib inhibits cell viability. PTC cells (10^4^) were exposed to increasing doses of volasertib for 24 and 48 h and cell viability was assessed using MTT. Data were presented as mean ± SD (*n* = 8). (D, E) Cell cycle analysis of PTC cells treated with volasertib. BCPAP and TPC‐1 cells were exposed to 250 and 500 nm volasertib for 48 h. Following incubation, either cells were analyzed for cell cycle fractions by flow cytometry (D) or proteins were extracted from cells and were subjected to immuno‐blotting to analyze cell cycle markers (E). Data were presented as mean ± SD (*n* = 3). (F, G) Volasertib induces apoptosis in PTC cell lines. PTC cells were exposed to indicated doses of volasertib for 48 h. Following incubation, either cells were stained with annexin‐V/PI and analyzed by flow cytometry (F) or proteins were extracted from cells and were subjected immuno‐blotting using antibodies as indicated (G). Data were presented as mean ± SD (*n* = 3). (H) Effect of volasertib on Bax activation in PTC cells. PTC cells were treated with volasertib for indicated time course. Following treatment, cells were lysed in 1% Chaps lysis buffer and subjected to immuno‐precipitation with anti‐Bax antibody (sc‐493; Santa Cruz) and immunoblotted with anti‐Bax antibody (B8554; SIGMA‐ALDRICH), (*n* = 3). Statistical analyses were conducted employing two‐tailed Student's *t*‐tests. *P < 0.05.

The PLK1 overexpression was observed in our PTC cohort and was significantly correlated with aggressive clinicopathological parameters. Hence, our objective was to explore whether inhibiting PLK1 could serve as a feasible therapeutic approach to impede cell growth and trigger apoptosis in PTC cells. Initially, we assessed PLK1 protein expression in Nthy‐ori 3–1, a normal human primary thyroid follicular epithelial cell line, and other three PTC cell lines (BCPAP, TPC‐1, and K1). Our analysis revealed elevated PLK1 expression in two PTC cell lines (BCPAP and TPC‐1), while K1 and Nthy‐ori 3–1 cell lines displayed low levels of PLK1 expression (Fig. 2A). Subsequently, we treated escalating doses of volasertib to PTC cell lines, BCPAP and TPC‐1, and assessed cell viability using the MTT assay after 24 and 48 h of treatment. As shown in Fig. 2B,C, there was a significant dose‐dependent inhibition of cell viability after 48 h in both cell lines. We selected volasertib doses 250 and 500 nm and 48 h time point for further *in vitro* experimentation.

To discern whether the reduction in cell viability was attributed to cell cycle arrest or apoptosis, we treated PTC cells with volasertib (250 and 500 nm) for 48 h, followed by analysis for cell cycle and apoptosis. As anticipated, our findings demonstrated that volasertib treatment resulted in a reduction in the G1 phase of the cell cycle, subsequently leading to G2/M phase arrest and apoptosis (Fig. 2D). We also analyzed the expression levels of important cell cycle proteins regulated by PLK1.

The volasertib treatment reduced the levels of cyclin B1, Cyclin D1, and phopho‐CDK1 in these cells (Fig. 2E). To confirm apoptosis in volasertib‐treated PTC cells, we treated BCPAP and TPC‐1 cells with volasertib for 48 h, and stained with annexin V and propidium iodide and analyzed for apoptosis by flow cytometry. As shown in Fig. 2F, there was an increase in apoptotic cells after treatment with volasertib in both the PTC cell lines tested. We also examined the expression of apoptotic protein markers after volasertib treatment on these cells by immunoblotting. The volasertib treatment induced the cleavage of PARP and caspase‐3 in these cells (Fig. 2G). In addition, treatment with volasertib caused an increase in the total Bad and a decrease in phosphorylated Bad as well as Bcl‐2 protein expression in these cells (Fig. 2G).

The treatment of volasertib at different time points also showed Bax protein conformational changes at 8 h in both PTC cell lines (Fig. 2H).

We additionally overexpressed PLK1 in Nthy‐ori 3–1 and evaluated the cell growth by clonogenic assay. As shown in Fig. S1A,B, forced expression of *PLK1* significantly increased cell growth. In addition, we examined whether overexpression of PLK1 affects the distribution of cells in the different cell cycle phases (Fig. S1C). We noted a substantial rise in the proportion of cells residing in the S phase, which is the most actively proliferative phase, in Nthy‐ori 3–1 cells with PLK1 overexpression. These results indicate that the upregulation of PLK1 increases the cell proliferation *in vitro*. On the contrary, the knockdown of *PLK1* significantly decreased the cell growth in BCPAP and TPC‐1 cells (Fig. S1D,E).

### 3.5 PLK1 interacts directly with FoxM1 *in vitro*

**Fig. 3:**
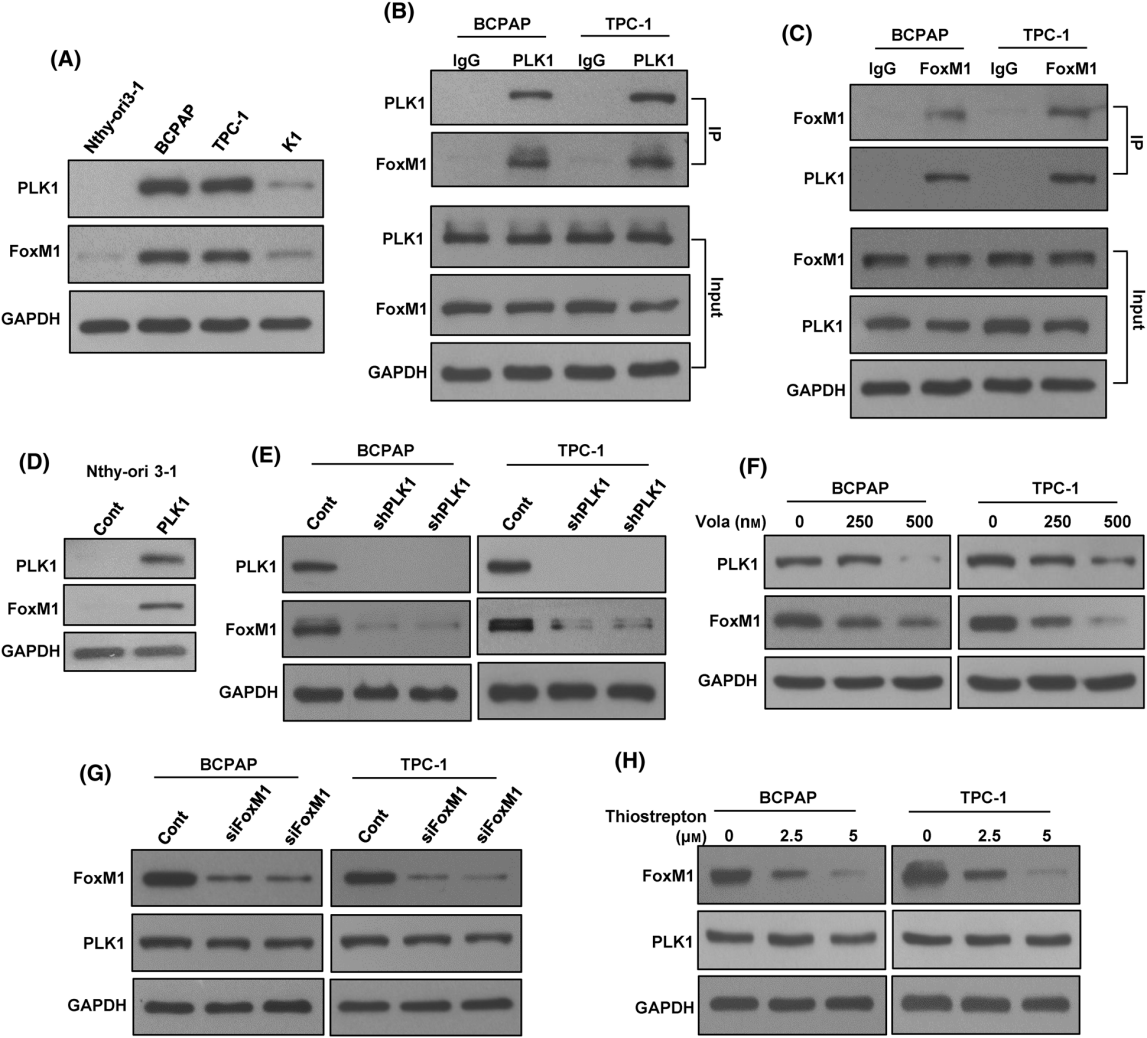
PLK1 interacts with FoxM1 *in vitro*. (A) Basal expression of PLK1 and FoxM1 in PTC cell lines. Proteins were isolated from Nthy‐ori 3–1 cell line and three PTC cell lines and immunoblotted with antibodies against PLK1, FoxM1, and GAPDH (*n* = 3). (B) PLK1 interact with FoxM1. Cell lysates extracted from PTC cells were immunoprecipitated with PLK1 or IgG antibody. Interaction of endogenous PLK1 and FoxM1 was detected by immunoblotting (*n* = 3). (C) FoxM1 interact with PLK1. Cell lysates extracted from PTC cells were immunoprecipitated with FoxM1 or IgG antibody. Interaction of endogenous FoxM1 and PLK1 was detected by immunoblotting (*n* = 3). (D) Forced expression of *PLK1* triggers the activation of FoxM1 expression. Nthy‐ori 3–1 cells were transfected with either an empty vector or PLK1 cDNA for 48 h. Proteins were extracted from overexpression clones and were subjected immunoblotting using antibodies as indicated (*n* = 3). (E) Knockdown of *PLK1* inhibits FoxM1. PTC cells underwent transfection with two distinct PLK1 shRNA sequences, and proteins from the selected clones were later analyzed using immunoblotting (*n* = 3). (F) Volasertib treatment reduce the expression of PLK1 and FoxM1 in PTC cells. PTC cells were treated with specified doses of volasertib for 48 h. Proteins were extracted after cell lysis and were subjected to immunoblotting, as indicated (*n* = 3). (G, H) Inhibition of FoxM1 has no effect on PLK1 expression. PTC cells were either transfected with FoxM1 siRNA (20 nm) or treated with thiostrepton (2.5 and 5 μm). Following 48 h, the cells were lysed, and the extracted proteins were subsequently subjected to immunoblotting using antibodies as indicated (*n* = 3).

Our clinical data revealed a significant correlation between PLK1 and FoxM1 in PTC cases (Table 2). To investigate this interaction *in vitro*, we initially evaluated the expression of PLK1 and FoxM1 in Nthy‐ori 3–1, BCPAP, TPC‐1, and K1 cell lines by western blotting. We found associated expression of PLK1 and FoxM1 in two PTC cell lines (BCPAP and TPC‐1), whereas cells with low or negligible expression of PLK1 showed low expression of FoxM1 (Fig. 3A). The physical interaction between PLK1 and FoxM1 was examined by immunoprecipitation (IP) analysis *in vitro*. The result shows that PLK1 binds to FoxM1 (Fig. 3B) and vice versa (Fig. 3C) in PTC cell lines.

To confirm the PLK1 and FoxM1 association *in vitro*, we stably overexpressed PLK1 in Nthy‐ori 3–1 cell line and examined the FoxM1 expression. As shown in Fig. 3D, forced expression of *PLK1* dramatically increased the FoxM1 expression in this cell line. Next, we stably knockdown *PLK1* using two separate sequences of shRNA's in both BCPAP and TPC‐1 cells and analyzed PLK1 and FoxM1 expression. Knockdown of *PLK1* markedly down‐regulated the expression of FoxM1 in these cells (Fig. 3E). Similar results were observed when PLK1 was inhibited using specific PLK1 inhibitor, volasertib (Fig. 3F). We also inhibited FoxM1 expression using specific siRNA or inhibitor to see the effect on PLK1 expression. As shown in Fig. 3G,H, inhibition of FoxM1 using either siRNA or specific inhibitor, thiostrepton successfully down‐regulated the expression of FoxM1, whereas PLK1 expression remained unchanged. All these results indicate the association of PLK1 with FoxM1 *in vitro*.

### 3.6 Combined inhibition of PLK1 and FoxM1 markedly attenuates PTC cell growth *in vitro*

**Fig. 4:**
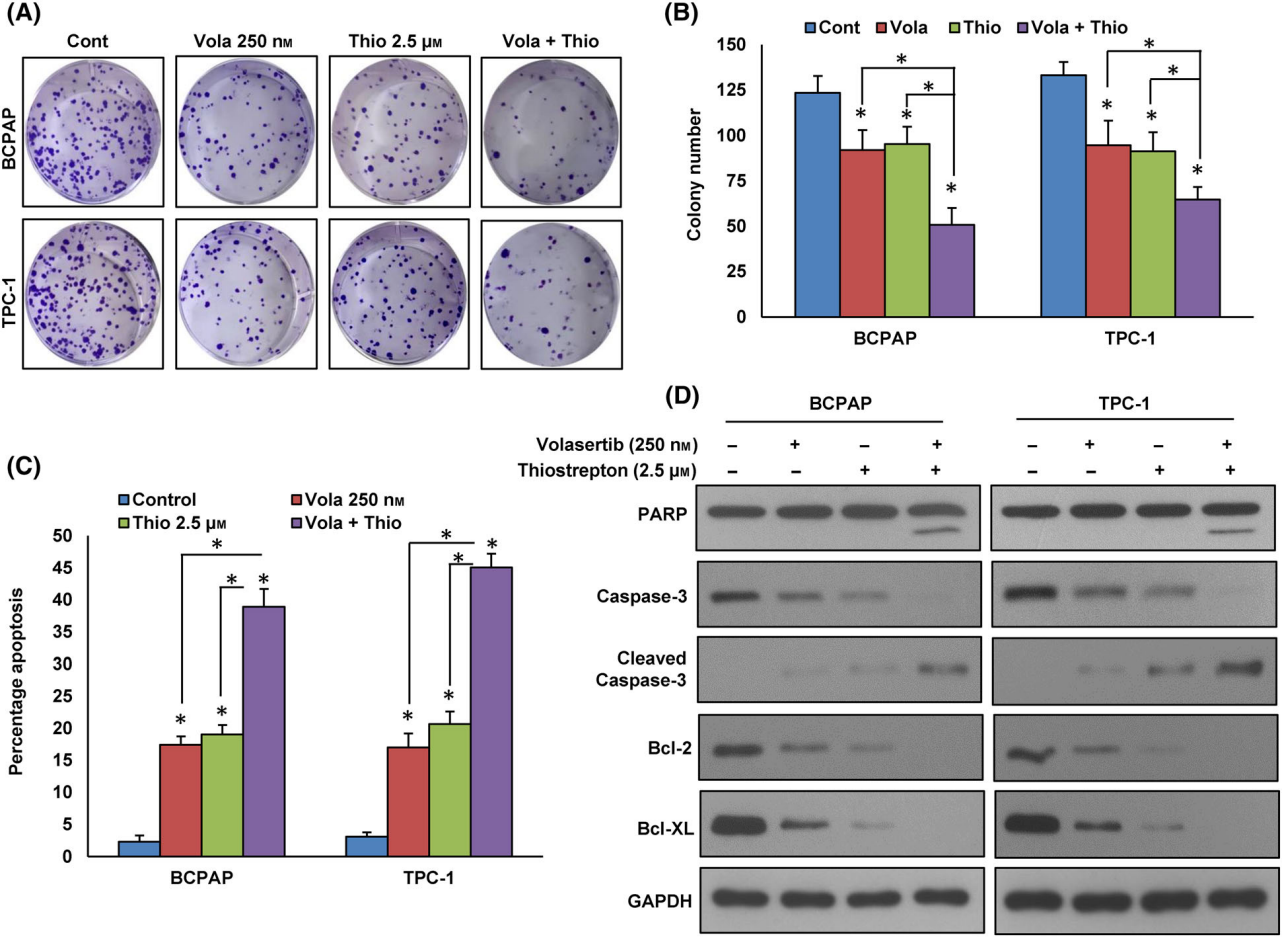
Combined inhibition of PLK1 and FoxM1 markedly attenuates PTC cell growth *in vitro*. (A–B) Volasertib and thiostrepton synergistically inhibits clonogenicity. PTC cells (5 × 10^2^) after volasertib and thiostrepton treatments were seeded into each of three dishes (60 mm diameter), and grown for an additional 10 days, then stained with crystal violet and colonies were counted. Data were presented as mean ± SD (*n* = 3). Statistical analyses were performed using two‐tailed Student's *t*‐tests. *P < 0.05. (C) Volasertib and thiostrepton synergistically induce apoptosis. PTC cells were exposed to specified doses either of volasertib and thiostrepton, single or in combination, for 48 h. Subsequently, these cells were stained with annexin‐V/PI and analyzed by flow cytometry. Data were presented as mean ± SD (*n* = 3). Statistical analyses were performed using two‐tailed Student's *t*‐tests. *P < 0.05. (D) Volasertib and thiostrepton synergistically induce the cleavage of caspase‐3 and PARP. PTC cells after 48 h of indicated treatment, lysed and proteins were subjected to immunoblot analysis using antibodies as indicated (*n* = 3).

We showed a strong association between PLK1 and FoxM1 protein expression in PTC cell lines and patient tissues. Therefore, we assume that combined inhibition of PLK1 and FoxM1 expression could induce an efficient cytotoxic effect in PTC cells. The combination of varying doses of volasertib with sub‐optimal dose of thiostrepton exhibited a synergistic reduction in cell viability in PTC (Fig. S2). Using calcusyn software [34], we determined that in the BCPAP cell line, a concentration of 250 nm volasertib combined with 2.5 μm thiostrepton resulted in a combination index (CI) of 0.673, indicating synergy (Fig. S3). In the TPC‐1 cell line, the same combination had a CI of 0.624, also indicating synergy in inhibiting cell viability (Fig. S4). We selected these specific doses for further experimental investigations. We showed that combination of volasertib and thiostrepton significantly (*P* < 0.05) decreased the colony number of PTC cells as compared to treatment alone (Fig. 4A,B). Furthermore, combination of volasertib and thiostrepton synergistically potentiated apoptosis in PTC cells (Fig. 4C). As shown in Fig. 4D, the combination of volasertib and thiostrepton also induced the cleavage of PARP and caspase‐3 as well as reduced the expression of anti‐apoptotic proteins, Bcl‐2 and Bcl‐xL in these cells.

### 3.7 Inhibition of PLK1 decreases the self‐renewal potential of spheroids formed from PTC cells

**Fig. 5:**
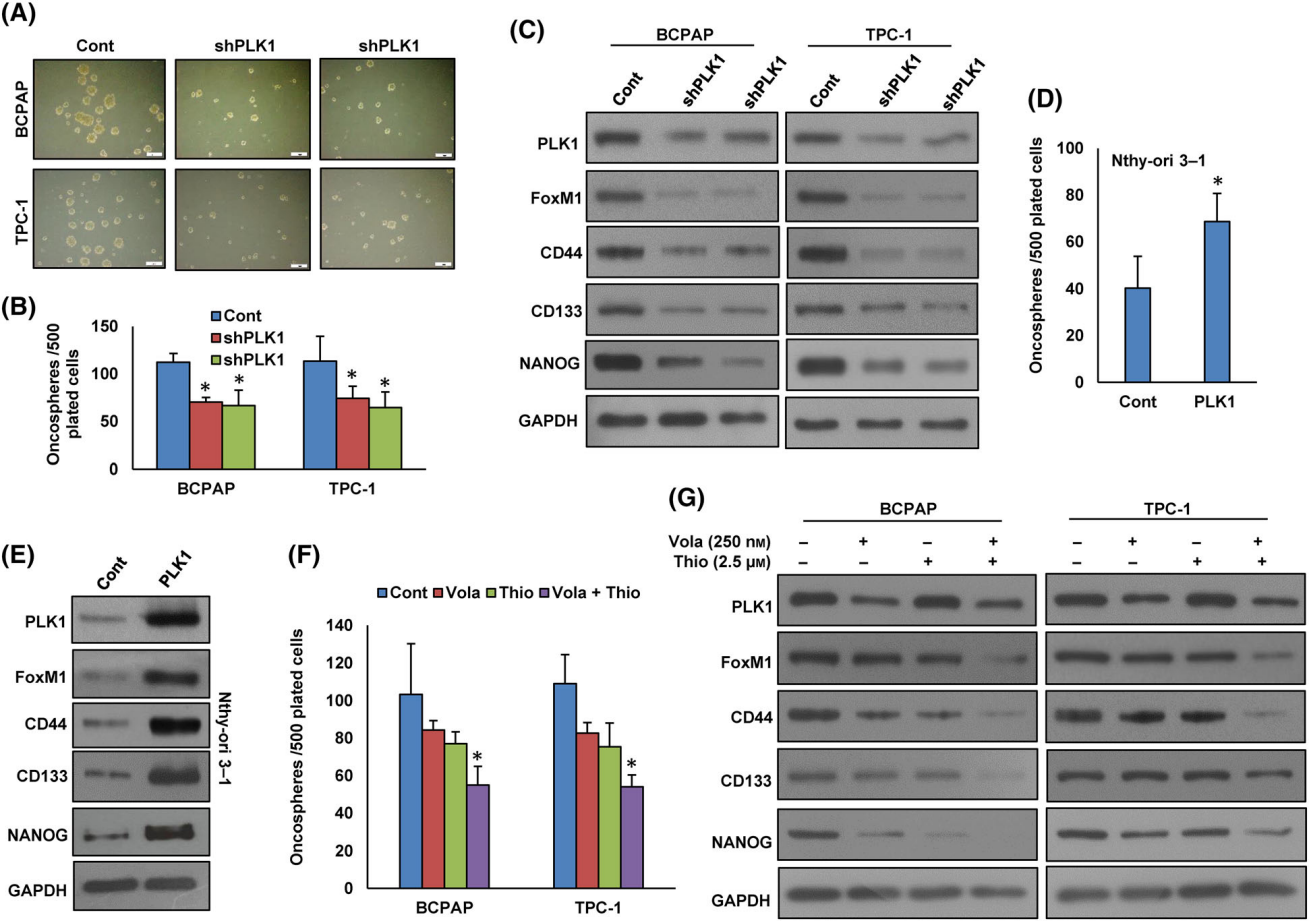
Inhibition of PLK1 decreases the self‐renewal ability of spheroids generated from PTC cells. (A, B) Knockdown of *PLK1* impairs the self‐renewal capacity of spheroids. PTC cells were transfected with PLK1 shRNA and subsequently subjected to a sphere‐forming assay (scale bar = 1 mm). The total number of spheroids in the entire well was quantified. Data were presented as mean ± SD (*n* = 3). (C) Knockdown of *PLK1* reduces the stem cell characteristics of spheroids. PTC cells were transfected with PLK1 shRNA and cultured in a sphere‐forming medium. Subsequently, proteins were extracted from the spheroids and analyzed via immunoblotting using antibodies as indicated (*n* = 3). (D) Forced *PLK1* expression enhances the growth of spheroids. Nthy‐ori 3–1 cells were transfected with either an empty vector or PLK1 cDNA, and subsequently, these cells were subjected to a sphere‐forming assay. The total number of spheroids in the entire dish was quantified. Data were presented as mean ± SD (*n* = 3). (E) Forced expression of *PLK1* enhances the stem cell properties of spheroids, as validated through immunoblotting employing stem cell markers. Nthy‐ori 3–1 cells were transfected with either an empty vector or PLK1 cDNA and subsequently cultured in a sphere‐forming medium. Proteins were isolated from spheroids and immunoblotted with antibodies against PLK1, FoxM1, CD44, CD133, NANOG, and GAPDH (*n* = 3). (F) Volasertib and thiostrepton synergistically reduces spheroid growth. PTC cells were exposed to specified doses of volasertib and thiostrepton, either single or in combination, for 48 h. Following this treatment, the cells were subjected to a sphere‐forming assay. Spheroids in the entire dish were counted. Data were presented as mean ± SD (*n* = 3). (G) PTC cells were exposed to specified doses either of volasertib and thiostrepton, single or in combination, for 48 h. Subsequently, these treated cells were cultured in a sphere‐forming medium and proteins isolated from spheroids were subjected to immunoblotting using antibodies as indicated (*n* = 3). Statistical analyses were performed using two‐tailed Student's *t*‐tests. *P < 0.05.

A recent study demonstrates that PLK1 plays a critical role in maintaining cancer stemness [35]. To explore the role of PLK1 in spheroid growth in PTC, we established stable *PLK1* knockdown PTC cell lines and cultured them in spheroid medium. Intriguingly, the knockdown of *PLK1* led to a significant reduction in spheroid growth (Fig. 5A,B) and a downregulation in the expression of stem cell markers such as CD133, CD44, and NANOG (Fig. 5C). Moreover, the forced expression of *PLK1* in the Nthy‐ori 3–1 cell line resulted in enhanced spheroid growth (Fig. 5D) and an increased expression of PLK1, FoxM1, CD44, CD133, and NANOG, compared to cells transfected with an empty vector (Fig. 5E). Furthermore, our findings demonstrate that the combined inhibition of PLK1 and FoxM1, using volasertib and thiostrepton, respectively, led to a significant reduction in spheroid growth (Fig. 5F) and a decrease in the stemness properties (Fig. 5G) of both PTC cell lines.

### 3.8 Combined inhibition of PLK1 and FoxM1 delays PTC tumor growth *in vivo*

**Fig. 6:**
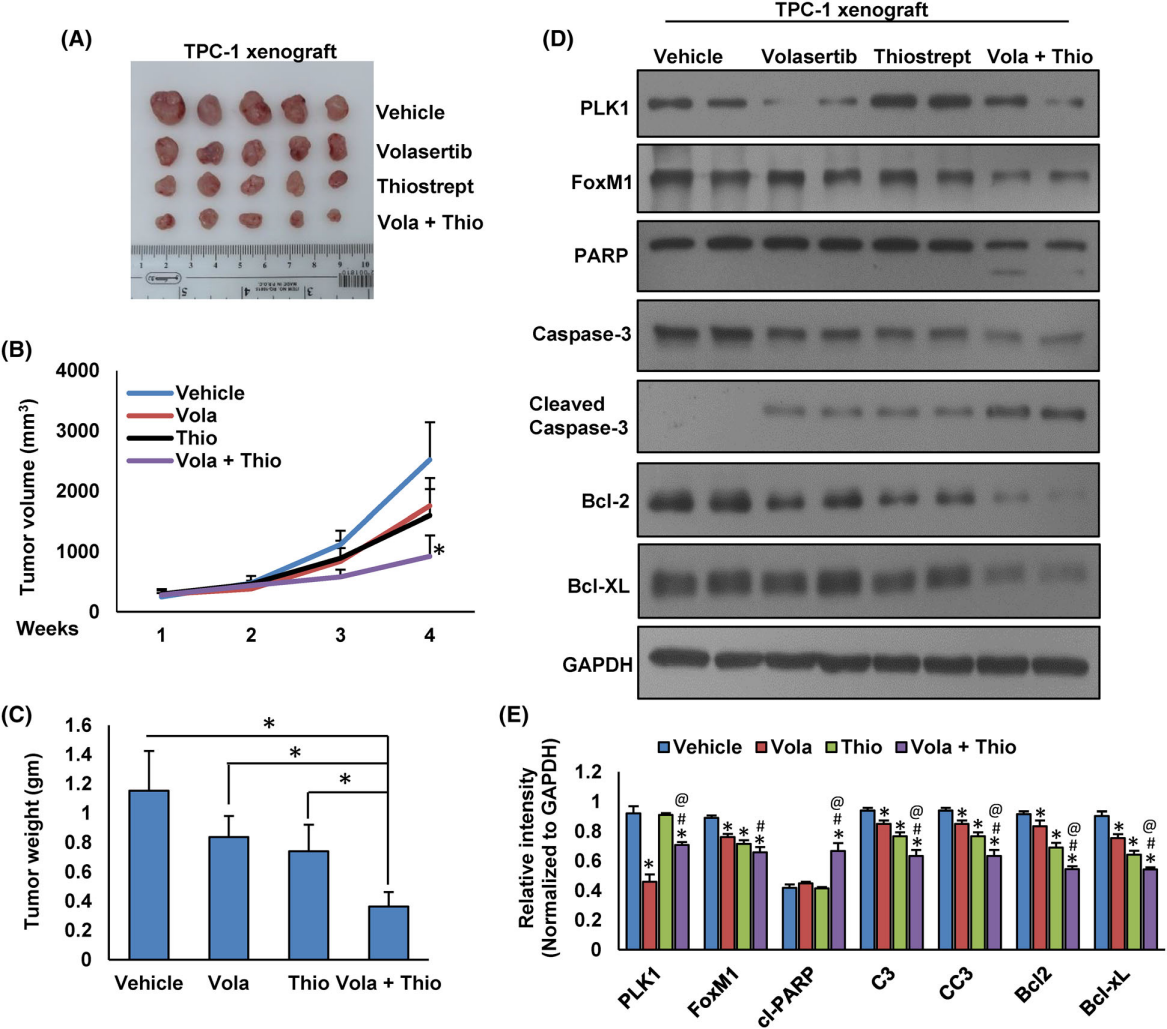
Volasertib and thiostrepton synergistically inhibit PTC tumor growth *in vivo*. TPC‐1 cells were subcutaneously injected into the flanks of 6‐week‐old NU/J mice (4 × 10^6^ cells per mouse). Once the tumors reached approximately 100 mm^3^ in size, the mice were subjected to intraperitoneal treatment with the specified doses of volasertib (20 mg·kg^−1^) and thiostrepton (20 mg·kg^−1^), either administered individually or in combination. This treatment regimen was repeated twice a week and continued for a duration of 30 days. (A) Representative images of tumors from each group of mice. (B) The tumor volume measurements were taken every week. The average (*n* = 5) tumor volume (±SD) in each group of mice was calculated, *P < 0.05. (C) Following 4 weeks of treatment, the mice were euthanized, and the mean tumor weight (±SD) was determined for each group, *P < 0.05. (D) Tissue lysates from tumors were immuno‐blotted with antibodies against PLK1, FoxM1, PARP, Caspase‐3, Cleaved‐caspase‐3, Bcl‐2, Bcl‐xL, and GAPDH. (E) Western blots were quantified and data are the mean ± SD (*n* = 3). Statistical analyses were performed using two‐tailed Student's *t*‐tests. *Statistically significant difference compared to vehicle control with *P* < 0.05. ^#^Statistically significant difference compared to volasertib treatment with *P* < 0.05. ^@^Statistically significant difference compared to thiostrepton treatment with *P* < 0.05.

We showed that synergistic inhibition of PLK1 and FoxM1 significantly suppressed PTC cell growth *in vitro*. We also investigated whether the combined administration of volasertib and thiostrepton could delay the growth of PTC tumors in NU/J mice. In the xenograft study, we inoculated TPC‐1 cells (4 × 10^6^ cells per mouse) into the flanks of 6‐week‐old female NU/J mice. After the tumors had developed (about 100 mm^3^), the mice were injected (i.p) with volasertib (20 mg·kg^−1^) and thiostrepton (20 mg·kg^−1^) either alone or in combination, twice a week for 30 days. DMSO (0.1%, i.p) was served as vehicle control. We found that co‐treatment of volasertib with thiostrepton delayed tumor growth as shown by reduced tumor size and volume (Fig. 6A,B) as well as tumor weight (Fig. 6C) but had no effect on the body weight of mice (data not shown). Further, co‐treatment of volasertib with thiostrepton markedly suppressed the expression of PLK1, FoxM1 and induced cleavage of PARP and caspase‐3 as well as downregulation of Bcl‐2 and Bcl‐xL expression in tumor tissues (Fig. 6D,E). These data clearly demonstrate that combined inhibition of PLK1 and FoxM1 augment antitumor effects in TPC‐1 cell xenografts in NU/J mice.

## 4 Discussion

PLK1 dysregulation has been reported in different tumor types and its overexpression has been associated poor patients' outcome, resistance to chemotherapy and radiotherapy [11, 36, 37, 38]. However, the role of PLK1 in PTC pathogenesis is unclear. In this study, we reveal that PLK1 is highly expressed in 54.2% of PTC tumor sample (874/1612). Elevated PLK1 expression has been notably correlated with several aggressive clinicopathological factors, including extrathyroidal extension, advanced stage, lymph node metastasis, and tumor recurrence.

Interestingly, PLK1 overexpression was associated with important cell regulator FoxM1 which has been shown previously by us [18] and others [39] to play an important role in Middle Eastern PTC aggressiveness. However, after adjusting for clinical factor, a multivariant COX proportional hazard model indicated that only PLK1 is able independently to serve as independent marker for recurrence free survival.

Inhibition of PLK1 in PTC cell lines with volasertib caused G2/M phase arrest and mitotic block. Our study provides several lines of evidence supporting the oncogenic role of PLK1 in PTC. First, inhibition of PLK1 reduces PTC cell growth *in vitro* and *in vivo*. Second, our *in vitro* data show a potential role of PLK1 in regulation of cancer‐stem cell‐like cells in PTC. Inhibition of PLK1 significantly suppressed the spheroid growth and downregulated the expression of stem cell markers such as CD44, CD133, NANOG in PTC. This shows that PLK1 might have a potential role in maintaining stemness properties in PTC.

The significant association between PLK1 and FoxM1 overexpression in the study cohort is not surprising. PLK1 and FoxM1 overexpression has been observed in a variety of several types of cancer [16, 40, 41]. However, their relationship within PTC had not fully illustrated. Here, we have demonstrated that both PLK1 and FoxM1 are overexpressed in PTC samples and cell lines. Our research extends to targeting two interrelated and mutually reinforcing pathways (PLK1 and FoxM1) within PTC cells, aiming to achieve a more effective anti‐proliferative response. The concurrent inhibition of these pivotal pathways has not been previously explored in PTC. In our study, we combined volasertib and thiostrepton in PTC cell lines, resulting in a synergistic anti‐proliferative effect and inhibition of cell growth observed in both *in vitro* and *in vivo* experiments.

Another interesting finding of this study is the identification of regulatory relationship between PLK1 and FoxM1 in PTC. Our data show that PLK1 co‐immunoprecipitated with FoxM1 in PTC. In addition, ectopic expression of PLK1 increase FoxM1 expression whereas PLK1 inhibition decrease FoxM1 expression. Interestingly, FoxM1 inhibition has no effect on PLK1 expression, suggesting that PLK1 functions via FoxM1, which is in line with previous studies, where PLK1 positively regulates FoxM1 in different tumor types [16, 39]. The above finding suggests the importance of using therapeutic interventions that focuses on targeting PLK1‐FoxM1 mediated signaling network.

The strategy of combining therapeutic targets may offer enhanced efficacy, particularly in specific aggressive subtypes of thyroid cancer. Our study places emphasis on targeting the intertwined pathways (PLK1 and FoxM1) in PTC, with the goal of achieving a superior anti‐proliferative response. In PTC cell lines, we observed a synergistic anti‐proliferative effect when inhibiting both PLK1 and FoxM1, leading to reduced cell viability and the induction of apoptosis. These results were further supported by *in vivo* studies where the combination of both volasertib and thiostrepton therapy significantly delayed xenograft tumor growth when compare with either single agent or control treatment.

## 5 Conclusion

Taken together, our study showed that PLK1 plays an important prognostic role in Middle Eastern PTC. We also found promising therapeutic effect of volasertib and thiostrepton combination in the treatment of PTC tumors, in particular, the subset of patient that overexpress FoxM1. These findings can potentially expand the clinical applications of volasertib in the treatment of PTC. The studies involving combination with FoxM1 inhibitor hold significant translational potential, paving the way for novel therapeutic approaches in the treatment of thyroid cancer.

## Conflict of interest

The authors declare no conflict of interest.

## Author contributions

PKP and AKS designed, performed experiments, and wrote the manuscript. SKP prepared the TMA and conducted all the immunohistochemistry experiments and scoring of IHC spots. DP, ST, KA, RD, RB, and WH performed experiments. FA‐D and SSA‐S collected and analyzed all the clinical samples and data. KSA‐K made substantial contributions to conception, design, and acquisition of data along with analysis and interpretation of data; prepared and wrote the manuscript. KSA‐K gave the final approval for the submission of the manuscript. This is to confirm that all authors read and approved the final manuscript.

### Peer review

The peer review history for this article is available at https://www.webofscience.com/api/gateway/wos/peer‐review/10.1002/1878‐0261.13610.

## Supporting information


**Fig. S1.** PLK1 promotes cell growth *in vitro*.
**Fig. S2.** Synergistic effect of volasertib and thiostrepton on PTC cell viability.
**Fig. S3.** Synergistic inhibition of cell viability by volasertib and thiostrepton in BCPAP cells.
**Fig. S4.** Synergistic inhibition of cell viability by volasertib and thiostrepton in TPC‐1 cells.

## Data Availability

All data generated or analyzed during this study are included in this published article.
