# Supplementary material for: PLK1 and FoxM1 expressions positively correlate in papillary thyroid carcinoma and their combined inhibition results in synergistic anti‐tumor effects
Source: Mol Oncol. 2024 Feb 15;18(3):691–706. doi: 10.1002/1878-0261.13610 (PMC10920088; doi:10.1002/1878-0261.13610)
Supplement: Supplementary file 1 — Fig. S1. PLK1 promotes cell growth in vitro. Fig. S2. Synergistic effect of volasertib and thiostrepton on PTC cell viability. Fig. S3. Synergistic inhibition of cell viability by volasertib and thiostrepton in BCPAP cells. Fig. S4. Synergistic inhibition of cell viability by volasertib and thiostrepton in TPC‐1 cells. [file MOL2-18-691-s001.zip › mol213610-sup-0005-Supplementary_Legends.docx]

**Supporting Information section**

**Supplementary Figure 1:** **PLK1 promotes cell growth *in vitro*. (A-B)** Forced expression of *PLK1* increases clonogenicity. Nthy-ori 3-1 cells were transfected with either an empty vector or *PLK1* cDNA for 48 h. Selected clones were seeded at a density of 500 cells per well in a 6-well plate, and grew for an additional 10 days, then stained with crystal violet, and colonies were counted. Data were presented as mean ± SD (n = 3). **(C)** Nthy-ori 3-1 cells after stable overexpression with PLK1 were stained with propidium iodide (PI) followed by flow cytometry analysis. Data were presented as mean ± SD (n = 3). **(D-E)** Knockdown of *PLK1* attenuates clonogenicity. PTC cells were transfected with PLK1 shRNA and selected clones were subjected to a clonogenicity assay. Data were presented as mean ± SD (n = 3). Statistical analyses were performed using two-tailed Student's t-tests. *p < 0.05.

**Supplementary Figure 2: Synergistic effect of volasertib and thiostrepton on PTC cell viability.** BCPAP and TPC-1 cells were incubated with indicated doses of volasertib and thiostrepton in combination for 48 hours. Cell viability was performed using MTT. Data were presented as mean ± SD (n = 8). Statistical analyses were performed using two-tailed Student's t-tests. *p < 0.05.

**Supplementary Figure 3:** Synergistic inhibition of cell viability by volasertib and thiostrepton in PTC cells. BCPAP cells were treated with various combinations of volasertib and thiostrepton for 48 h and dose effect (A) and Fractional effect (B) graphs were generated using Calcusyn software. (C) Combination Index (CI) were calculated using Chou and Talalay method.

**Supplementary Figure 4:** Synergistic inhibition of cell viability by volasertib and thiostrepton in PTC cells. TPC-1 cells were treated with various combinations of volasertib and thiostrepton for 48 h and dose effect (A) and Fractional effect (B) graphs were generated using Calcusyn software. (C) Combination Index (CI) were calculated using Chou and Talalay method.
