# Supplementary figures and images for: PLK1 and FoxM1 expressions positively correlate in papillary thyroid carcinoma and their combined inhibition results in synergistic anti‐tumor effects
Source: Mol Oncol. 2024 Feb 15;18(3):691–706. doi: 10.1002/1878-0261.13610 (PMC10920088; doi:10.1002/1878-0261.13610)

Supplementary figure 1

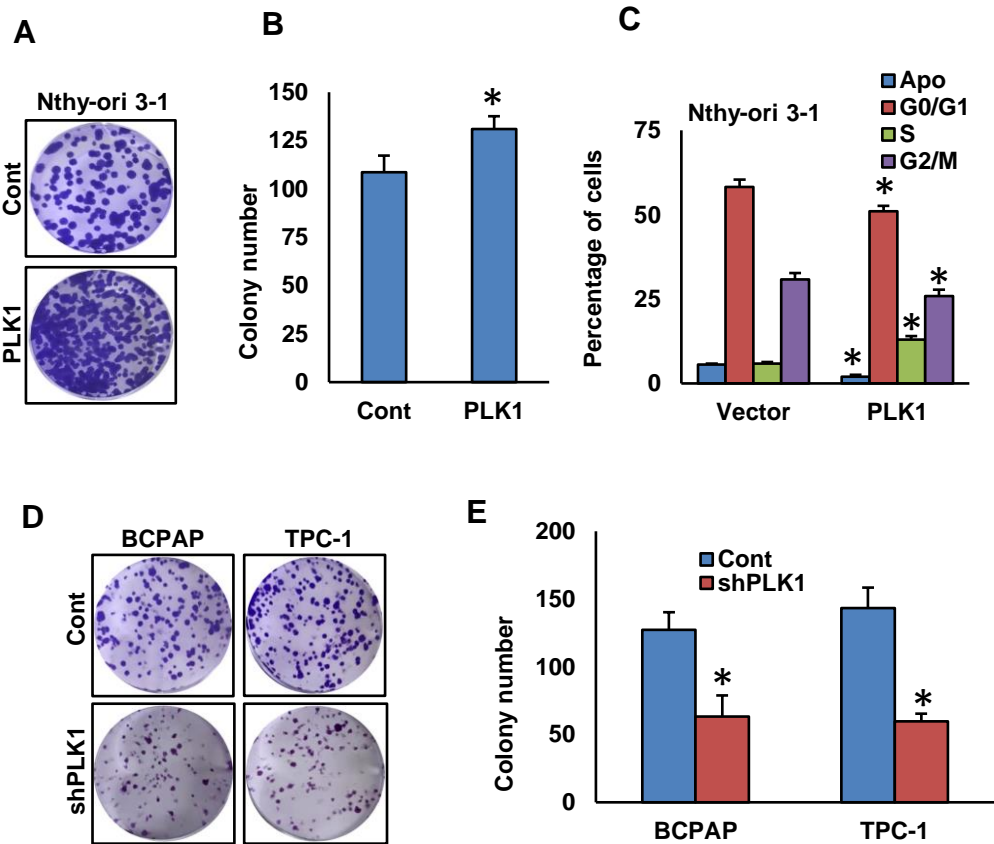

Supplement: Supplementary file 1 — Fig. S1. PLK1 promotes cell growth in vitro. Fig. S2. Synergistic effect of volasertib and thiostrepton on PTC cell viability. Fig. S3. Synergistic inhibition of cell viability by volasertib and thiostrepton in BCPAP cells. Fig. S4. Synergistic inhibition of cell viability by volasertib and thiostrepton in TPC‐1 cells. [file MOL2-18-691-s001.zip › mol213610-sup-0001-FigS1.pdf]

Supplementary figure 2

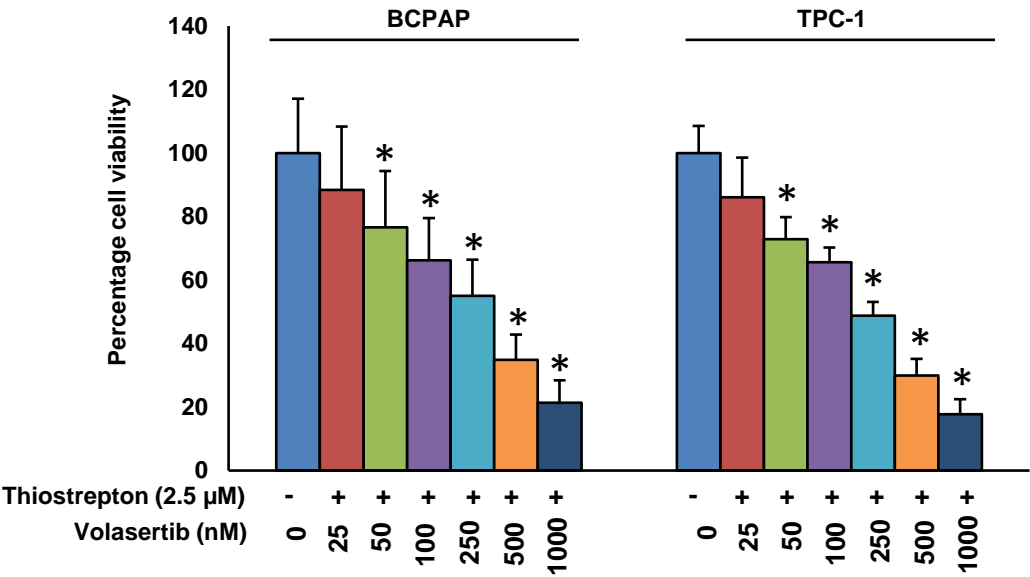

Supplement: Supplementary file 1 — Fig. S1. PLK1 promotes cell growth in vitro. Fig. S2. Synergistic effect of volasertib and thiostrepton on PTC cell viability. Fig. S3. Synergistic inhibition of cell viability by volasertib and thiostrepton in BCPAP cells. Fig. S4. Synergistic inhibition of cell viability by volasertib and thiostrepton in TPC‐1 cells. [file MOL2-18-691-s001.zip › mol213610-sup-0002-FigS2.pdf]
